# The structure of an endogenous *Drosophila* centromere reveals the prevalence of tandemly repeated sequences able to form i-motifs

**DOI:** 10.1038/srep13307

**Published:** 2015-08-20

**Authors:** Miguel Garavís, María Méndez-Lago, Valérie Gabelica, Siobhan L. Whitehead, Carlos González, Alfredo Villasante

**Affiliations:** 1 Centro de Biología Molecular “Severo Ochoa” (CSIC-UAM), Universidad Autónoma de Madrid, Nicolás Cabrera 1, 28049 Madrid, Spain; 2 Instituto de Química Física Rocasolano, CSIC, Serrano 119, 28006 Madrid, Spain; 3 Univ. Bordeaux, ARNA Laboratory, IECB, 2 rue Robert Escarpit, F-33600 Pessac, France; 4 Inserm ARNA Laboratory, 146 rue Leo Saignat, F-33000 Bordeaux, France; 5 The Wellcome Trust Sanger Institute, Hinxton, Cambridgeshire, United Kingdom

## Abstract

Centromeres are the chromosomal loci at which spindle microtubules attach to mediate chromosome segregation during mitosis and meiosis. In most eukaryotes, centromeres are made up of highly repetitive DNA sequences (satellite DNA) interspersed with middle repetitive DNA sequences (transposable elements). Despite the efforts to establish complete genomic sequences of eukaryotic organisms, the so-called ‘finished’ genomes are not actually complete because the centromeres have not been assembled due to the intrinsic difficulties in constructing both physical maps and complete sequence assemblies of long stretches of tandemly repetitive DNA. Here we show the first molecular structure of an endogenous Drosophila centromere and the ability of the C-rich dodeca satellite strand to form dimeric i-motifs. The finding of i-motif structures in simple and complex centromeric satellite DNAs leads us to suggest that these centromeric sequences may have been selected not by their primary sequence but by their ability to form noncanonical secondary structures.

Centromere sequences evolve rapidly due to inevitable recombination processes undergone by tandemly repeated sequences but many centromere proteins are conserved^1^. This paradox could be explained by the presence of a conserved sequence-independent structural motif, rather than a particular sequence motif^2^. The maintenance of a centromere-specific motif through evolution could be driven by molecular co-evolution of centromeric DNA and centromeric proteins. Importantly, in support of this evolutionary hypothesis, it has been shown that the centromere-specific histone H3 variant CENP-A (CID in *Drosophila*) has evolved in concert with centromeric satellite DNAs^3^,^4^.

Although a centromeric structural motif might be sufficient to direct the formation of centromeric chromatin on its own, the episodic occurrence of centromere activity associated with noncentromeric sequences, neocentromeres^5^,^6^, and the frequent inactivation/reactivation of centromeres^7^,^8^,^9^,^10^,^11^,^12^ indicates that centromere specification involves both genomic competency and epigenetic mechanisms^13^. It is now recognized that CENP-A-containing nucleosomes provide the epigenetic mark to establish the centromere-specific chromatin^14^,^15^,^16^, and that the centromeric chromatin contains blocks of CENP-A nucleosomes interspersed with blocks of canonical histone H3 nucleosomes^17^. However, the folding of this centromeric chromatin is still not clear and is of much controversy^18^,^19^.

Although there is evidence to suggest that each *Drosophila melanogaster* endogenous centromere is made up of different simple and complex satellite DNAs^20^,^21^,^22^,^23^,^24^,^25^, their molecular structure has yet to be determined. The centromeric region of chromosome 3 of *D. melanogaster*, as well as the centromeric region of chromosomes 2 and 3 of the sibling species *Drosophila simulans* and *Drosophila mauritiana*, contains dodeca satellite 11/12 bp tandem repeats (CCCGTACTGGT/CCCGTACTCGGT) showing asymmetric distribution of guanine and cytosine residues such that one strand is predominantly G-rich and the other C-rich^25^,^26^,^27^.

In order to fully understand the structural and functional aspects of centromeres, it is important to elucidate the types of secondary DNA structures that can be formed by their constituent repeat units. Hence, we determined that the G-rich dodeca satellite strand is able to fold into very stable intramolecular hairpin structures that are stabilized by the formation of noncanonical G:A pairs^28^, and recently we have shown that not only the type B monomer of the human centromeric alpha-satellite^28^ but also the type A are able to form dimeric i-motif structures^29^. The i-motif is a four-stranded intercalated structure formed by the association of two parallel duplexes combined in an antiparallel fashion by forming intercalated hemi-protonated C:C^+^ base pairs^30^,^31^,^32^,^33^. As i-motif formation requires protonation of cytosines these structures are more stable at acidic pH, although, depending on particular C-rich sequences, they can fold at neutral pH^34^,^35^. I-motifs can also exist at neutral pH under molecular crowded conditions^36^ and under transcriptionally induced negative superhelicity^37^.

Since centromere specification may rely on centromeric structural motifs under the control of epigenetic mechanisms, knowledge of the fine structure of the endogenous *D*. *melanogaste*r centromeres is required to elucidate the potential formation of noncanonical centromeric DNA structures. However, due to the repetitive nature of centric heterochromatin, the endogenous centromeres remain poorly represented in the new *D*. *melanogaster* Release 6 reference genome sequence^38^.

The heterochromatin of *D. melanogaster* has been subdivided into 61 distinct cytological regions^39^, and the primary constriction of the third chromosome localizes asymmetrically within the h53 region^39^. Previous work has shown that the dodeca satellite DNA hybridized very close to the primary constriction of the third chromosome, but extending to the right arm^26^, and that, occasionally, two hybridization signals can be seen in prometaphase chromosomes^27^. Moreover, the cytological analysis of the free chromosome arm *F(3R)1* has shown that the amount of dodeca satellite on the right arm can be reduced without compromising chromosome segregation^26^. Initial studies of the long-range structure of the dodeca satellite DNA were reported by^27^. Common-cutting restriction enzymes that do not cut within dodeca satellite DNA revealed that most of the dodeca satellite was organized in two major blocks. In addition, the distribution of restriction sites in the long-range map suggested that the region between the blocks consists of complex DNA sequences, while the flanking region of one of the blocks is likely to contain another putative block of repetitive DNA yet undescribed.

## Results and Discussion

**Figure 1:**
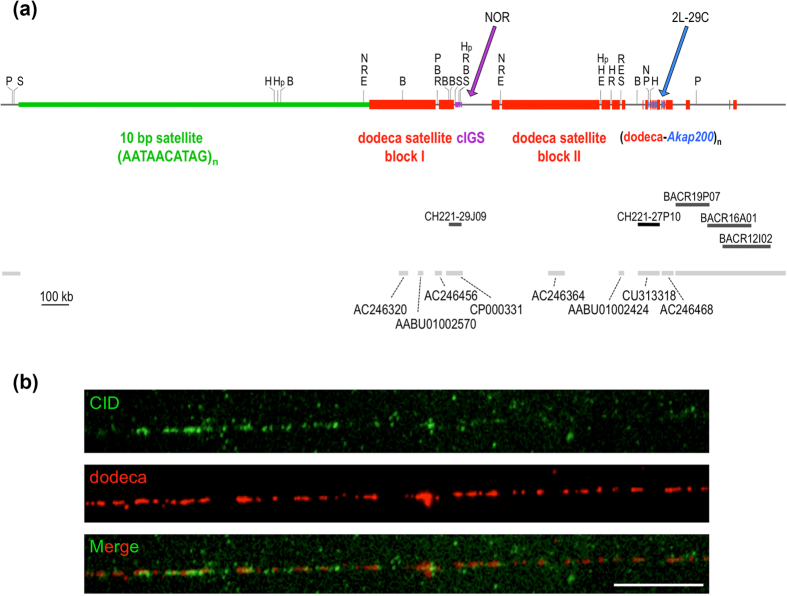
Structure of the centromere of the third chromosome of *D*. *melanogaster.* (**a**) 2.5 Mb physical map across the centromere of chromosome 3. The regions containing the 10 bp satellite repeats and the dodeca satellite repeats appear in green and red, respectively. The segmental duplications from the NOR and from region 2L-29C are indicated in purple and blue, respectively. The position and GenBank number of the eight centromeric scaffolds are also indicated. Abbreviations are: B, *BamHI*; H, *BssHII*; E, *BstEII*, R, *EcoRI*; N, *NaeI*; Hp, *HpaI*; P, *PmeI*, S, *SwaI*. (**b**) Extended chromatin fibers from S2 cells were processed for immunofluorescence with an anti-CID antibody followed by FISH with the dodeca satellite oligo probe. A representative image showing CID immunostaining (green) overlapping with dodeca (red) is shown. Of a total of 43 chromatin fibers stained with the anti-CID antibody, 14 showed co-localization with dodeca, a proportion in agreement with the karyotype of the polyploid S2 cells. CID signals do not encompass all dodeca satellite repeats. A minor number of fibers containing dodeca satellites are not stained with the anti-CID antibody. Scale bar is 5 μm.

Since a complete physical map across the centromere should extend from chromosome arm 3L to chromosome arm 3R, we set out to isolate bacterial artificial chromosome (BAC) clones that contain dodeca satellite, and to construct a comprehensive map around the dodeca satellite blocks using 20 restriction enzymes that do not occur within the dodeca satellite. Single and double genomic digests were size-fractionated by pulsed-field gel electrophoresis (PFGE) using a “Waltzer” apparatus^40^, which gives sharp resolution up to 2 Mb (representative digests are shown in Supplementary Fig. S1). In order to obtain dodeca satellite clones, three *D. melanogaster* BAC libraries were screened: the RPCI-98 library generated by cloning EcoRI-digested genomic DNA and the CHORI-221 and CHORI-223 libraries generated from sheared genomic DNA. BAC end sequencing and fingerprinting data of the stronger clones were used to construct contigs, and five clones were chosen for complete sequencing: CH221-29J09 (containing rDNA intergenic spacer (IGS) related sequences, cIGS), CH211-27P10 (containing *Akap200* related sequences) and BACR19P07, BACR16A01 and BACR12I02 that were also positive for the retrotransposon *Circe*. The presence of *Circe* sequences in the centromeric region h53 had previously been reported^41^. By combining data from the sequence of these BACs and the sequence of whole genome shotgun scaffolds containing dodeca satellite with the results of an accurate restriction site mapping of genomic DNA, it has been possible to determine the position and orientation of the first eight scaffolds in the Release 6 assembly of the chromosome arm 3R (Fig. 1a).

The dodeca satellite sequences at this endogenous centromere are organized as two adjacent major blocks, block I and block II, plus several minor blocks (Fig. 1a). Interestingly, a detailed analysis of the sequence of the blocks has shown that block I has more undeca than dodeca repeats (Supplementary Fig. S2) and that the sequence of both repeat units are highly conserved (Supplementary Fig. S3). Moreover, this centromeric region contains transposable elements and two segmental duplications: one results from a duplication of a fragment of *Akap200* (chromosome arm 2L at 29C) and subsequent amplification, and the other, located at one edge of block I, results from a duplication of IGS sequences at the nucleolus organizer region (NOR) (Fig. 1a). Nevertheless, FISH to mitotic chromosomes under low-stringency conditions with a cIGS-specific probe has not detected IGS-related sequences in the centromeric region of chromosome 2, although clear cross-hybridization signals occur at the NORs (Supplementary Fig. S4).

To elucidate whether CID interacts with dodeca satellite sequences, immunofluorescence-FISH experiments were performed. Thus, by using *SuUR Su(var)3–9* double mutants to suppress the normal under-replication of *Drosophila* heterochromatin during the process of polytenization, we showed that CID co-localizes on polytene chromosomes with dodeca satellite sequences^42^. To corroborate this interaction, we increased the resolution performing immunofluorescence-FISH on extended chromatin fibers from cultured S2 cells and found that anti-CID antibody and dodeca satellite signals co-localize in approximately one fourth of the CID-positive fibers (n = 46), consistent with dodeca satellite being present only in the centromeric heterochromatin of chromosome 3, and not in the other four *Drosophila melanogaster* chromosomes (Fig. 1b). Some of the fibers that were positive for dodeca did not show co-staining for CID. This could indicate that dodeca satellite, most likely block II and minor blocks, extends beyond the centromeric chromatin, which contains CID nucleosomes. A similar scenario has been observed in the centromeres of *Arabidopsis thaliana*, in which part of the 178 bp satellite repeats extend beyond the centromeric chromatin into pericentromeric regions. This pericentromeric 178-bp satellite associates with H3.1-containing chromatin, while the centromeric 178-bp repeats associate with CENH3-containing chromatine^43^,^44^. Although the assembly of *Droshophila melanogaster* centromere 3 presented here is not completed at a base pair level, our results strongly suggest that dodeca-satellite block I is a good candidate for the centromere of chromosome 3.

**Figure 2:**
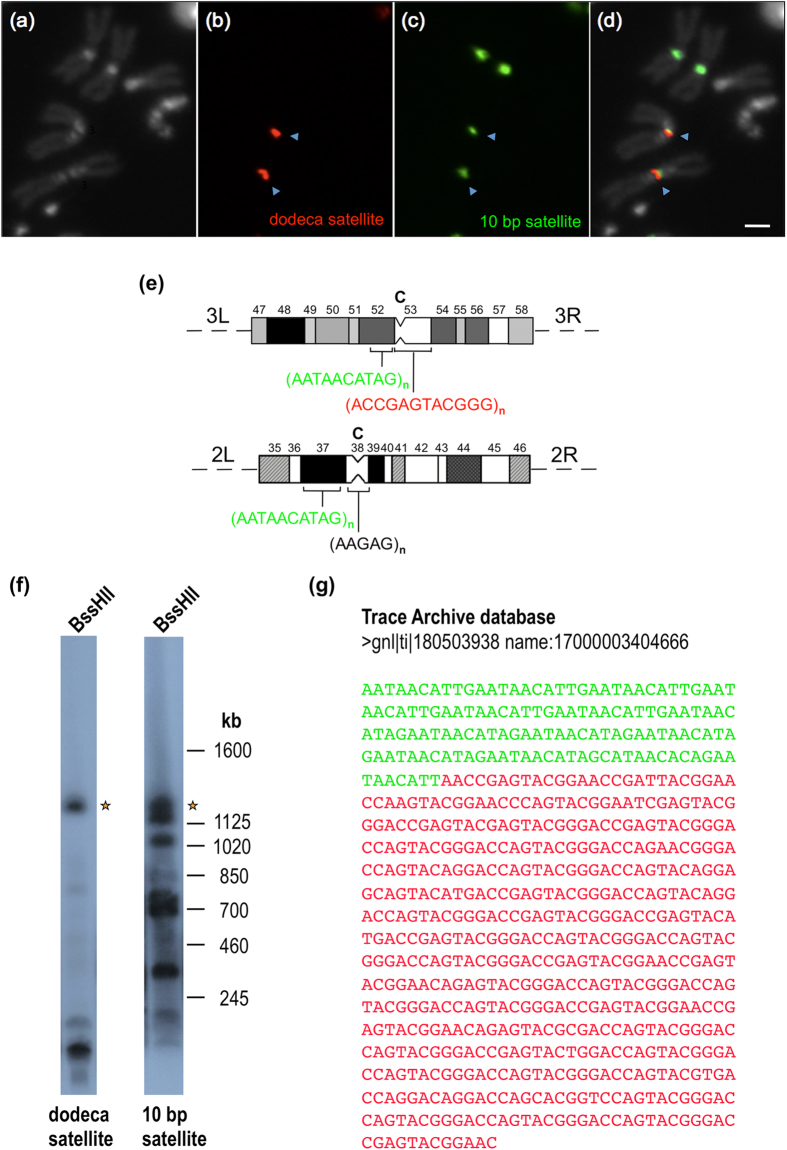
The 10 bp satellite DNA localizes on the third chromosome at h52p instead of h48. (**a**) Metaphase chromosomes counterstained with DAPI. (**b**) Hybridization signals from a dodeca satellite probe (in red). (**c**) Hybridization signals from a 10 bp satellite probe (in green). (**d**) Hybridization signals superimposed with DAPI-stained chromosomes. The Scale bar is 2 μm. (**e**) Diagram representing the heterochromatic regions^39^ of chromosomes 2 (regions 35–46) and 3 (regions 47–58) showing the localization of the 10 bp (in green) and dodeca (in red) satellites. The position of the centromeres (C) is indicated. (**f**) High molecular weight DNA from *red e* embryos was digested with *BssHII*, electrophoresed through a 1% (w/v) agarose gel in a “Waltzer” apparatus at 150 V for 24 h with a 130 s pulse time, blotted onto a nylon filter and hybridized successively with the dodeca satellite probe pBK6E218 at 68 °C and with the 10 bp satellite probe 5′-AATAACATAGAATAACATAGAATAACATAGAATAACATAGAATAACATAG-3′ at 50 °C. The asterisks indicate a 1.2 Mb fragment that hybridizes with both probes. (**g**) DNA sequence showing a junction between 10 bp satellite repeats (in green) and dodeca satellite repeats (in red).

During our effort to identify the putative block of simple sequence DNA in the flanking region of the dodeca satellite block 1, we repeated the cytological mapping of the 10 bp satellite (AATAACATAG)n using a fluorescent probe, which improves sensitivity and resolution with respect to results obtained with tritiated probes^22^. The 10 bp satellite had been mapped by^22^ to region h37 on the second chromosome (contiguous to the centromeric region h38) and to region h48 on the third chromosome (far away from the centromeric region h53). Unexpectedly, FISH experiments with dodeca and 10 bp satellite probes revealed no additional sites for the 10 bp satellite, but showed a change in its location on the third chromosome from h48 to h52p, a position which is very close to dodeca satellite (Fig. 2a–e). To investigate further the possibility that the flanking satellite DNA corresponds to the 10 bp satellite, we asked whether the 1.2 Mb *BssHII* fragment containing both dodeca satellite and flanking satellite sequences would hybridize with the 10 bp satellite probe. To this end, genomic DNA was digested with *BssHII*, size-fractionated by PFGE, transferred to a nylon membrane, hybridized with the dodeca satellite probe and then stripped and re-hybridized with the 10 bp satellite probe. As can be seen in Fig. 2f, there is a 1.2 Mb fragment (labeled with an asterisk) that hybridizes with both probes. Finally, the junction between 10 bp satellite and dodeca satellite sequences was found by searching the Trace Archive database (Fig. 2g). This result indicates that the 10 bp satellite DNA is physically linked to the dodeca satellite DNA. Here, it is important to remember that PROD, a protein required for centromere condensation^45^ and that specifically recognizes the 10 bp satellite^45^, is located near but not in the CID-containing chromatin^46^. Therefore, the physical map constructed comprises two adjacent chromatin domains with distinct functions. Although we have not completed the assembly at a base pair level, the sequence obtained from the five newly sequenced BAC clones, together with pre-existing contigs, with the identification of large blocks of several DNA satellites, and with the correct re-location of the 10 bp satellite from h48 to h52p, represent the most comprehensive physical map and assembly across the centromere of chromosome 3 of *Drosophila melanogaster*.

**Figure 3:**
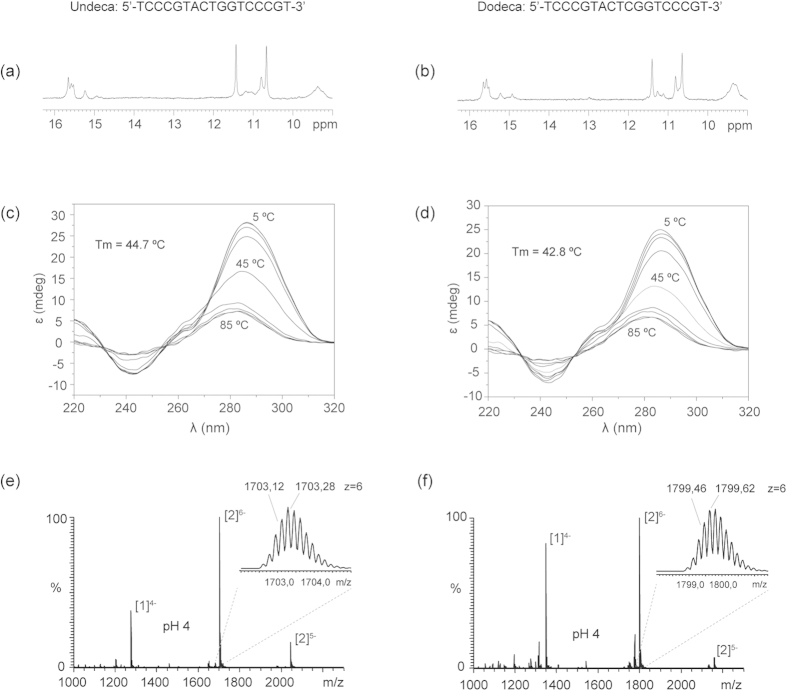
The centromeric dodeca satellite DNA is able to form dimeric i-motif structures. Imino region of the NMR spectra of the C-rich strands of the undeca (**a**) and dodeca repeats (**b**). Experimental conditions: Oligo concentration = 0.8 mM, 25 mM sodium phosphate, 100 mM NaCl, T = 5 °C, pH 4. CD spectra of the C-rich strands of undeca (**c**) and dodeca repeats (**d**) at different temperatures. Oligo concentration = 100 μM, same buffer as the NMR experiments. Mass spectrometry data showing the peaks of the single stranded [1] and dimeric [2] species formed by C-rich strands of undeca (**e**) and dodeca (**f**). Buffer conditions: 100 mM NH_4_OAc, spectra at pH 4. See Supplementary Fig. S7 legend for details.

**Figure 4:**
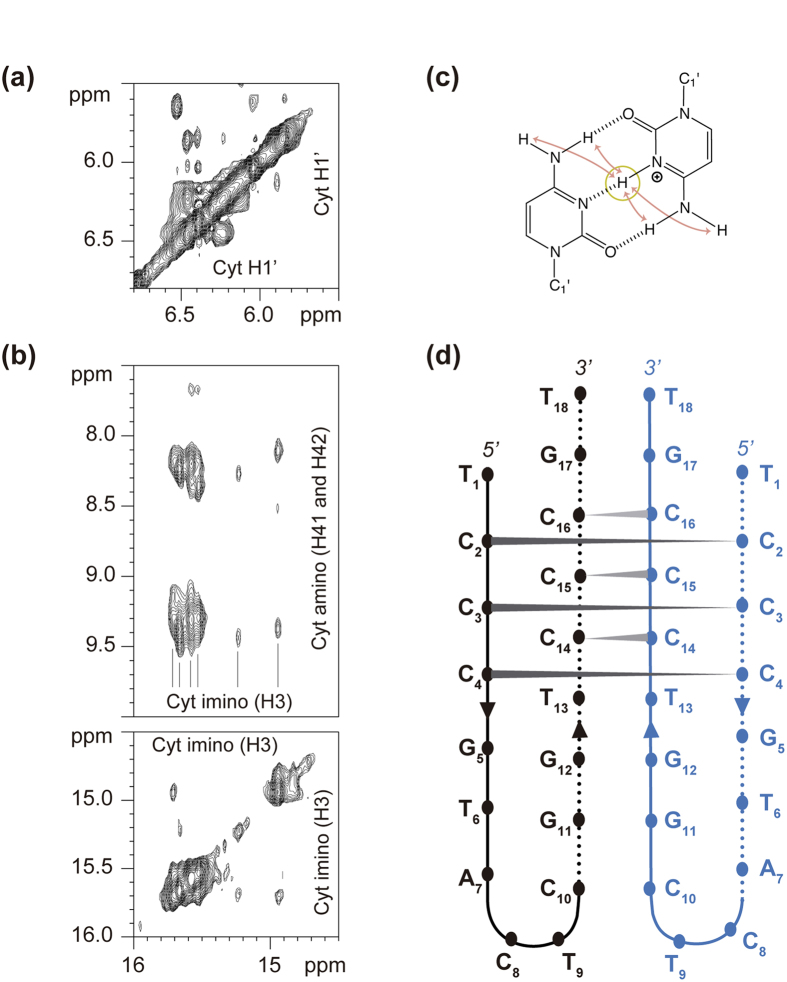
The dimeric i-motif structure of the centromeric dodeca satellite DNA is a head-to-head association of two hairpins. (**a**) Exchangeable proton region of the NOESY spectra. Each of the six cytosine imino signals exhibit two cross-peaks with cytosine amino protons, indicating the C:C^+^ base pairs occur between magnetically equivalent cytosines. (**b**) Region of the NOESY spectra of the C-rich strand of the dodeca repeat, showing characteristic H1’-H1’ cross-peaks (same experimental conditions as in Fig. 3b). (**c**) Scheme of a hemiprotonated C:C^+^ base pair, indicating the observable NOE cross-peaks between the cytosine imino and amino protons. (**d**) Schematic representation of the dimeric structure of C-rich strand of the dodeca repeat.

To determine the structural behavior of the dodeca satellite DNA, several oligonucleotides containing the dodeca repeat and, its main variant, the undeca repeat were studied by NMR, circular dichroism (CD) and mass spectrometry (MS). The G-rich and the C-rich strands were analyzed under different experimental conditions (Supplementary Fig. S5 and S6). In all cases, the NMR spectra of the G-rich oligonucleotides indicate the formation of G:C base pairs, and no formation of G-tetrads is observed even at high K^+^ concentrations (Supplementary Fig. S5). This is in agreement with the formation of intramolecular hairpins previously reported^47^. However, under acidic conditions the NMR spectra of the oligonucleotides corresponding to the C-rich strand of the dodeca and undeca repeats exhibit sharp imino signals around 15–16 ppm, characteristic of i-motif formation (Fig. 3a,b and Supplementary Fig. S6,8). I-motif formation is confirmed by CD spectra, which show the characteristic strong positive band at 285 nm^48^ (Fig. 3c,d). CD melting experiments show that these structures are quite stable at pH 4.0, with melting temperatures around 42 °C for dodeca and 45 °C for undeca (Supplementary Fig. S7e,f). Mass spectrometry data clearly indicate the formation of dimeric structures at acidic pH (Fig. 3e,f). The peaks corresponding to the dimeric species are not present at neutral pH and become very intense at pH 4 (Supplementary Fig. S7a–d). This pH dependence is consistent with i-motif structures. Interestingly, no tetrameric species are observed in mass spectrometry experiments, indicating that the structures formed by these oligonucleotides are the result of the self-association of two hairpins; similar dimeric structures as those observed in the A and B box of the human alpha satellite^28^,^29^. To further explore this similarity, we carried out two-dimensional NMR experiments of the dodeca oligonucleotide. Although a full structural determination is beyond the scope of this study, some interesting information can be readily spotted from the NOESY spectra. Each of the six cytosine imino signals (14.5–16.0 ppm) exhibit NOE cross-peaks with two amino protons (Fig. 4a). The presence of only two cross-peaks with cytosine amino protons instead of four (see Fig. 4c) denotes the formation of C:C^+^ base pairs between equivalent residues in each subunit. Other NOEs characteristic of i-motifs are also observed, such as imino-imino cross-peaks between adjacent C:C^+^ base pairs (Fig. 4a) or H1’-H1’ contacts between deoxyribose protons (Fig. 4b). According to these experimental data, we suggest a plausible structural model in which two hairpins self-associate in a head-to-head orientation through the formation of six intermolecular C:C^+^ base pairs (Fig. 4d).

In order to explore the existence of these structures under conditions that better represent a physiological context, we performed CD experiments at pH 6 and pH 7, and we explored the influence of the crowding agent PEG_4000_ on the stability of the structure. The CD spectra of both sequences at pH 7 show a maximum of ellipticity around 275 nm and a minimum around 240 nm (Supplementary Fig. S9). These CD spectra are different than those observed in single stranded DNA, and are considered as indicative of “i-motif-like” structures^49^. At pH 6, the maximum ellipticities of both sequences appear at around 285 nm, which is characteristic of i-motif structures. Importantly, the addition of 20% w/w PEG_4000_ produces a notable increase of the intensity at 285 nm (Supplementary Fig. S9), which indicates that the crowding conditions favour the formation of the i-motif at this pH. These results are totally consistent with previous observations on the stabilizing effect of crowding agents in other i-motif structures^36^, and suggest that the crowding environment present in the centromeric nucleosome might favour i-motif formation.

In conclusion, we have shown that the C-rich strand of the dodeca satellite (from both its 11 bp and 12 bp repeat units) is able to form dimeric i-motif structures *in vitro*. This experimental evidence, together with recent findings of similar i-motif structures in the human centromeric alpha-satellite^29^ lead us to suggest that we may be observing a structural motif common to centromeric sequences. Interestingly, preliminary results on the 359 bp satellite DNA (centromeric DNA from the Drosophila X chromosome)^22^ point in this direction, since the 359 bp C-rich region (found at internucleosomal linkers^50^) can also fold into i-motif structures (Supplementary Fig. S10).

These *in vitro* results stimulate the study of non-canonical DNA structures *in vivo*. The recent observation of G-quadruplex structures *in vivo* -20 years after their discovery *in vitro*- in telomeres and in gene promoters demonstrates that non-canonical DNA structures, different than the double helix, play significant roles in biological processes^51^. Much effort is still ahead to probe whether i-motifs exist *in vivo* or not, although recent findings on gene inhibition by small molecules that bind i-motif structures in promoter regions, suggest they might form *in vivo*^52^,^53^. If the occurrence of these structures were demonstrated in a centromeric nucleosomal context, they could be a structural signature of centromeric regions. Moreover, this would provide further support for our hypothesis that centromeric sequences are selected not by their primary sequence but by their ability to form noncanonical secondary structures^54^.

## Materials and Methods

### *Drosophila* strains and cell lines

Oregon R was used as wild-type strain. The isogenic *red e* strain was used for the construction of the physical map. Standard culture conditions and media were used. *Drosophila* S2 cells were grown and maintained as described^46^.

### DNA analysis, sequencing and probes

High molecular weight DNA from 0–12 h *Drosophila* embryos was prepared in agarose plugs as previously described by Ref. 25,55,56. Restriction enzyme digestions were performed following the suppliers’ recommendations. DNA was analyzed by pulsed-field gel electrophoresis using a “Waltzer” apparatus^40^, and transferred to Hybond N^+^ nylon filters (Amersham) in 0.4 M NaOH.

The dodeca satellite probe was pBK6E218^25^. The 10 bp satellite oligo probe was 5′-AATAACATAGAATAACATAGAATAACATAGAATAACATAGAATAACATAG-3′. The centromeric IGS (cIGS) probe (5.6 kb fragment) was obtained from BACR31J03 using the primers: cIGS-Fw: 5′-TGGCAGCGTTTTAAGGGATG-3′ and cIGS-Rv: 5′-TAAGACGCCTGCAGAGAACG-3′. The PCR was carried out as described by^57^. The PCR product was cloned in vector pGEM-T (Promega). Plasmid probes were ^32^P-labeled by random-priming and oligonucleotide probes were ^32^P-labeled with T4 polynucleotide kinase. The BAC clones were sequenced at The Wellcome Trust Sanger Institute by the standard shotgun sequencing and directed finishing approach. The GenBank accession number for the sequence of BAC19P07, BAC16A01, BAC12I02, CH221-29J09 and CH221-27P10 are CU311183, CR942806, CR942807, CU463787 and CU313318, respectively.

### Fluorescence *in situ* hybridization to mitotic chromosomes

Larval neuroblast chromosomes from Oregon R were prepared as described previously^58^. Chromosomes were counterstained with 4′,6-diamino-2-phenylindole (DAPI). The dodeca satellite oligo probe 5′-CCCGTACTGGTCCCGTACTGGTCCCGTACTCGGTCCCGTACTCGGT-3′ and the 10 bp satellite oligo probe 5′-AATAACATAGAATAACATAGAATAACATAGAATAACATAGAATAACATAG-3′ were chemically synthesized and labeled at the 5′ end with Cy3 or at the 3′ end with fluorescein (New England Biolabs). DNA probes derived from clones or PCR products were labeled by nick translation with digoxygenin-11-dUTP (Roche) using the DIG-Nick Translation Mix (Roche). Digoxygenin labeled probes were detected with Anti-Digoxigenin-Rhodamine, Fab fragments (Roche) in a 1:200 dilution, following supplier recommendations. Digital images were obtained using a Zeiss Axiover 200 microscope equipped with a cooled Charge-Coupled Device camera. The fluorescent signals were recorded separately as grey-scale digital images and then pseudo-colored and merged using Adobe Photoshop software.

### Immunofluorescence-FISH on extended chromatin fibers

Extended chromatin fibers were prepared from S2 cells by centrifuging 5 × 10^4^ cells onto slides at 800 rpm for 4 min in a Cytospin 4 (Thermo Shandon, Pittsburgh, PA), and then slides were dipped into salt detergent lysis buffer (25 mM Tris, pH 7.5, 500 mM NaCl, and 1% Triton X-100) for 25 minutes, slowly and steadily removed using an in-house made device consisting of a modified EasyDip™ Slide Staining System connected to a peristaltic pump, and subsequently fixed in 4% paraformaldehyde (PFA) for 5 minutes. Slides were incubated in 1×PBST (1×PBS ± 0.05% Tween-20) for 15 minutes. Slides were dipped again in the former lysis dilution for 15 minutes, after which they were slowly and steadily removed. Slides were blocked in 1×PBS, 0.1% Triton X-100, 4% formaldehyde for 10 minutes at room temperature and washed for 5 minutes in 1x PBS before proceeding to immunostaining. Slides were blocked in goat serum (Zymed Laboratories) for 30 minutes and incubated overnight at 4 °C with a chicken anti-CID antibody^46^, diluted to 1:100 in blocking buffer. Slides were washed 3 times for 5 minutes in 1×PBST and incubated for 1 hour at 37 °C in Alexa 488 anti-chicken secondary antibody (Molecular probes). Slides were then washed 3 times in 1×PBST and 3 times in 1×PBS. After immunofluorescence with CID antibodies, slides were re-fixed in 4% formaldehyde for 15 minutes and then hybridized to the dodeca probe. For each slide, around 250 ng of dodeca probe were precipitated with 3 M Sodium Acetate and absolute ethanol, re-suspended in hybridization solution (50% formamide, 10% dextran sulfate, 2×SSC) and denatured for 10 min at 80 °C. Slides were incubated at 37 °C for 24 h.

### DNA sample preparation for NMR and MS experiments

Oligonucleotides were purchased from Integrated DNA Technologies, IDT, Coralville, IA, USA. Samples for NMR experiments were dissolved in 9:1 H_2_O/D_2_O. Buffer conditions: 25 mM sodium phosphate, 100 mM NaCl pH 4.0 for C rich sequences and 25 mM potassium phosphate, 100 mM KCl pH 7.0 for G rich sequences. The latter were previously annealed by heating at 90 °C for 5 minutes and cooling down to room temperature overnight.

Samples for MS experiments were dissolved at 100 μM in 100 mM NH_4_OAc buffer at pH 7 and pH 4. pH was adjusted by adding acetic acid and NH_3_ aliquots.

### NMR experiments

All NMR spectra were acquired in Bruker spectrometers operating at 600 and 800 MHz, equipped with cryoprobes and processed with the TOPSPIN software. A jump-and-return pulse sequence^59^ was employed to observe the rapidly exchanging protons in 1D H_2_O experiments. In most of the experiments in H_2_O, water suppression was achieved by including a WATERGATE module in the pulse sequence prior to acquisition. NOESY experiments were recorded at 5 °C, in 25 mM NaPi, pH 4.0 (9:1 H_2_O/D_2_O) buffer and with mixing time of 100 ms.

### Circular Dichroism spectroscopy

Circular dichroism spectra at different temperatures were recorded on a Jasco J-810 spectropolarimeter fitted with a thermostated cell holder. CD spectra were recorded in 25 mM sodium phosphate buffer, pH 4, with 100 mM NaCl (100 μM oligo concentration). CD melting curves were recorded at the wavelength of the larger positive band, 285 nm with a heating rate of 0.5 °C.min^−1^.

Experiments with PEG_4000_ were performed by preparing the sample in buffer 25 mM NaPi, 100 mM NaCl containing 20% w/w PEG_4000_. A volume of the sample is weighted and an amount of 20% of the measured weight is added as PEG_4000_ (Sigma-Aldrich) to the sample. Then pH is fixed by adding HCl and NaCl aliquots.

### Mass spectrometry

All ESI-MS experiments were carried out in the negative ion mode on an Exactive ESI-Orbitrap mass spectrometer (Thermo Scientific, Bremen, Germany). The ESI spray voltage and capillary voltage used were −2.75 kV and −20 V, respectively. The capillary temperature was set to 150 °C. Tube lens and skimmer voltage were fixed to 180 V and −10 V, respectively. Samples were injected at a flow rate of 4 μL min^−1^.

## Additional Information

**How to cite this article**: Garavís, M. *et al.* The structure of an endogenous ^Drosophila^ centromere reveals the prevalence of tandemly repeated sequences able to form i-motifs. *Sci. Rep.* **5**, 13307; doi: 10.1038/srep13307 (2015).

## Supplementary Material

Supplementary Information
