# Supplementary material for: The structure of an endogenous Drosophila centromere reveals the prevalence of tandemly repeated sequences able to form i-motifs
Source: Sci Rep. 2015 Aug 20;5:13307. doi: 10.1038/srep13307 (PMC4542561; doi:10.1038/srep13307)
Supplement: Supplementary Information [file srep13307-s1.pdf]

The structure of an endogenous *Drosophila* centromere reveals the prevalence of tandemly repeated sequences able to form i-motifs.

Miguel Garavís[1,2], María Méndez-Lago[1,6], Valérie Gabelica[3,4], Siobhan L. Whitehead[5], Carlos González[2] * and Alfredo Villasante[1] *

[1]Centro de Biología Molecular "Severo Ochoa" (CSIC-UAM), Universidad Autónoma de Madrid, Nicolás Cabrera 1, 28049 Madrid, Spain. [2]Instituto de Química Física Rocasolano, CSIC, Serrano 119, 28006 Madrid, Spain. [3]Univ. Bordeaux, ARNA Laboratory, IECB, 2 rue Robert Escarpit F-33600 Pessac, France. [4] Inserm ARNA Laboratory, 146 rue Leo Saignat, F-33000 Bordeaux, France. [5]The Wellcome Trust Sanger Institute, Hinxton, Cambridgeshire, United Kingdom.

[6]Present address: Centro Nacional de Análisis Genómico, Baldiri Reixac 4, 08028 Barcelona, Spain.

Figures mentioned in the main text:

Figure S1. Gel electrophoresis analysis of dodeca satellite DNA digestions.

Figure S2. Linear representation showing the enrichment of dodeca and undeca repeats in scaffolds from block I and block II.

Figure S3. Diagram showing the sequence conservation of dodeca and undeca within sequenced scaffolds.

Figure S4. I-FISH of cIGS and 10 bp satellite to prometaphase chromosomes.

Figure S5. ^{1}H NMR spectra of dodeca and undeca G-rich sequences.

Figure S6. Imino region of 1D-NMR spectra of dodeca and undeca C-rich sequences in NH_4^+ buffer and in Na^+ buffer at different temperature.

Figure S7. Mass spectrometry data of dodeca and undeca sequences at neutral and acidic pH. CD melting curves and Tm values of dodeca and undeca sequences at pH 4.

Figure S8. ^{1}H NMR spectra of mutated dodeca and undeca oligonucleotides at different temperature.

Figure S9. CD spectra of dodeca and undeca at pH 7 and pH 6 in presence and absence of PEG_{4000}.

Figure S10. ^{1}H NMR spectra of an oligonucleotide derived from 359 satellite DNA at acidic pH.

Supplementary Figures

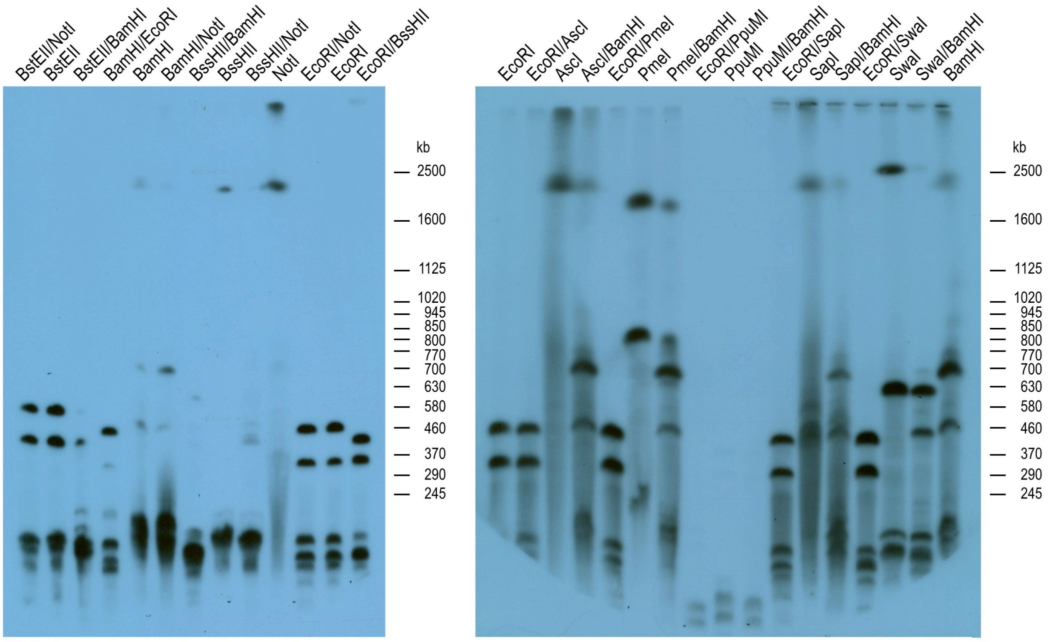

Figure S1. *Pulsed-field gel electrophoresis analysis of the dodeca satellite DNA. High molecular weight DNA from red e embryos was digested with one or two restriction enzymes, fractionated (using a "Waltzer" apparatus) on a 1.2% (w/v) agarose gel run at 150 V for 28 h with a pulse time of 80 s, blotted and hybridized with the dodeca satellite probe pBK6E218. Two representative gels are shown*.

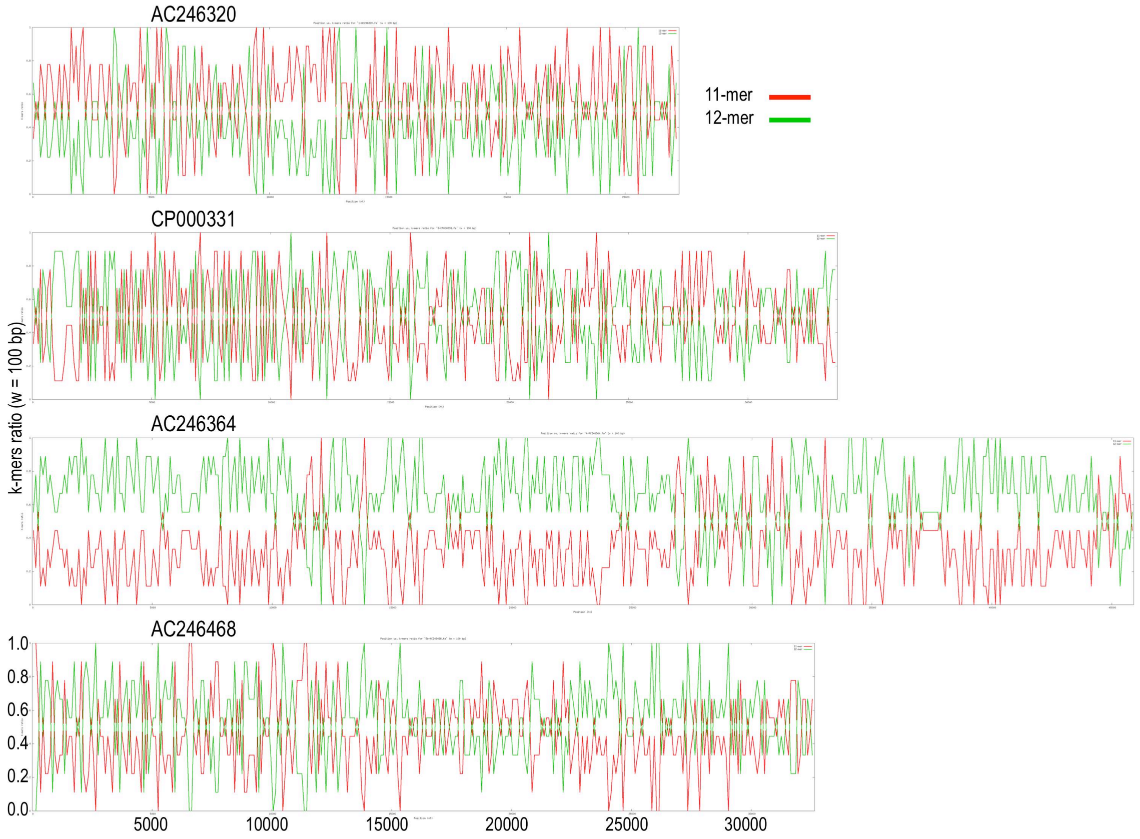

Figure S2. *Linear representation of undeca (in red) and dodeca (in green) repeats observed in two scaffolds from the dodeca satellite block I (AC246320 and CP00031), and two scaffolds from the dodeca satellite block II (AC246364 and AC246468).*

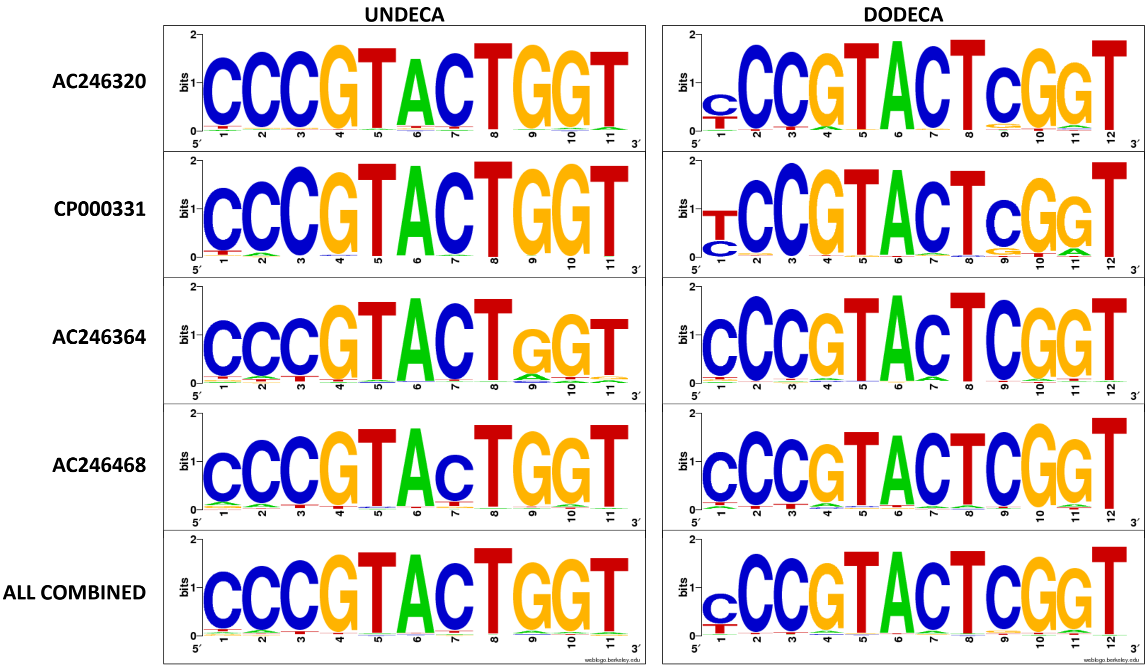

Figure S3. *Graphical representation of the sequence conservation of undeca and dodeca units present in four scaffolds (AC246320, CP000331, AC246364 and AC246468) and in the combination of all them generated with WebLogo[1]. Each logo consists of stacks of symbols, one stack for each position in the sequence. The overall stack height indicates the sequence conservation at that particular position, while the height of each symbol within the stack indicates their relative frequency.*

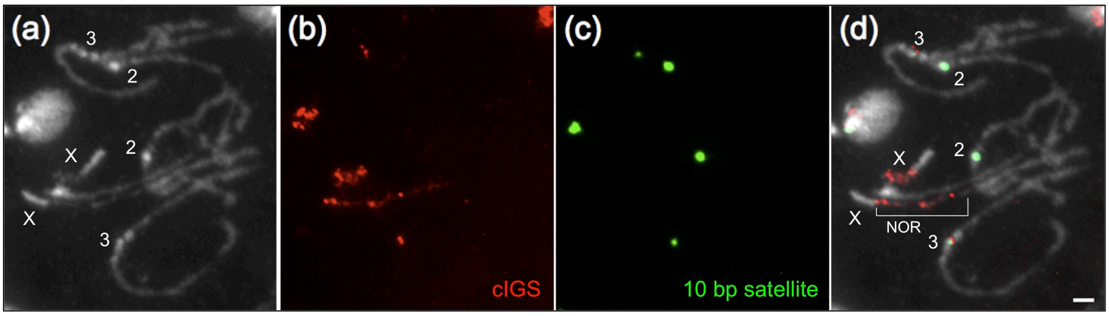

Figure S4. *Fluorescence in situ hybridization of cIGS and 10 bp satellite to prometaphase chromosomes from Oregon R females. The hybridization was performed under low-stringency conditions (25ºC). (a) Prometaphase chromosomes counterstained with DAPI. (b) Hybridization signals from a cIGS probe (in red). (c) Hybridization signals from a 10 bp satellite probe (in green). (d) DAPI stained superimposed with hybridization signals. The nucleolus organizer region (NOR) is indicated. The Scale bar is 2 µm.*

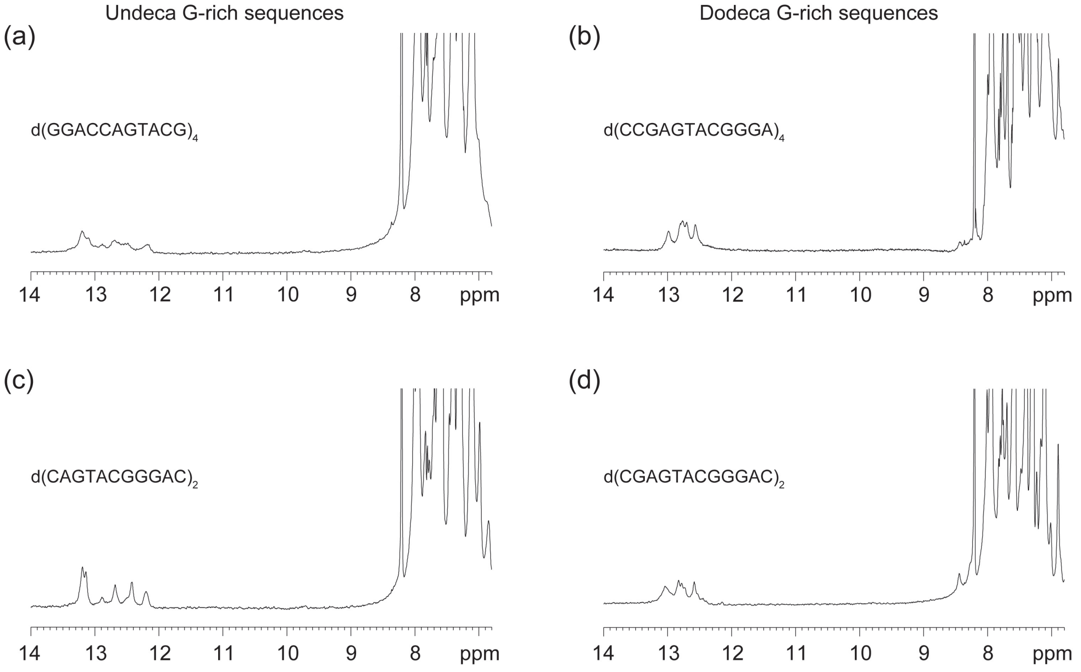

Figure S5. *Imino region of the NMR spectra of the G-rich stands. Top: oligos containing four repeats of the undeca sequences (a) and dodeca sequences (b). Bottom: Oligos containing two repeats of undeca (c) and dodeca (d). Experimental conditions: [oligo]=0.2 mM, 25 mM potassium phosphate, 100 mM KCl, T= 5ºC, pH 7.*

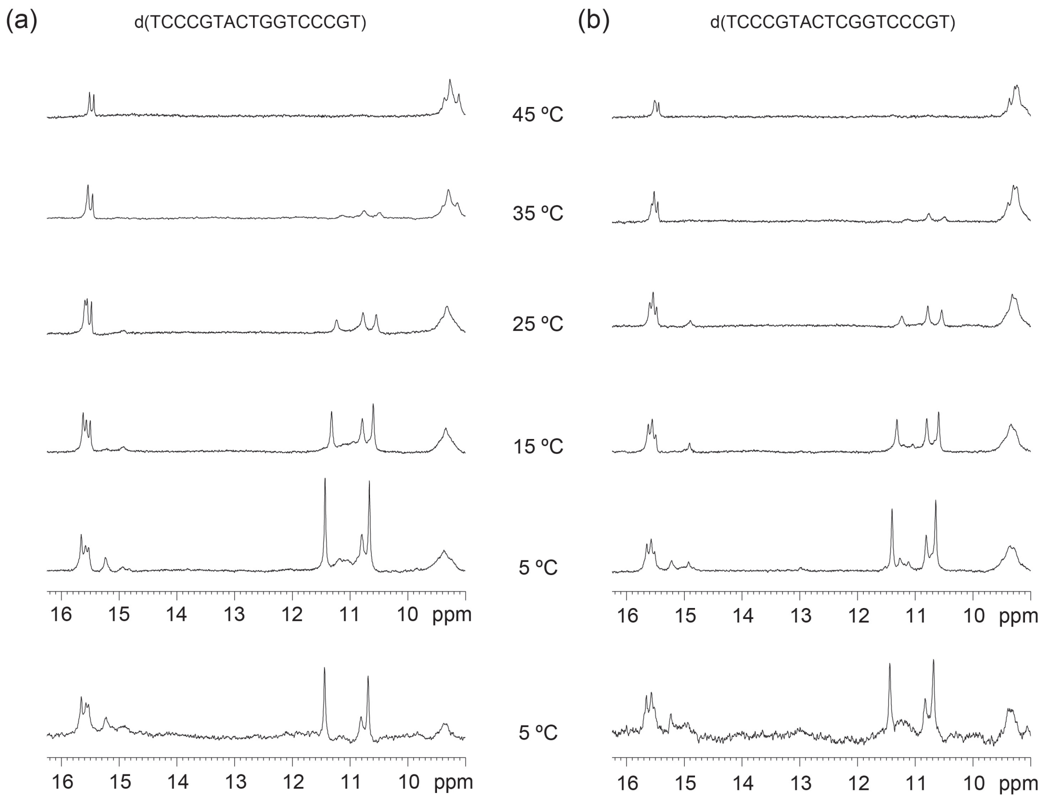

Figure S6. *Imino region of the NMR spectra of the C-rich strands of the undeca (a) and dodeca (b) at different temperatures. The signals around 15-16 ppm correspond with imino protons involved in hemiprotonated C:C$^+$ base pairs. See Supplementary Fig S8 for analogous experiments with control sequences. Experimental conditions: [oligo]=1.5 mM, 25 mM sodium phosphate, 100 mM NaCl, pH 4. Bottom spectra: 100 mM NH_4OAc, [oligo]=100 μM, T= 5ºC, pH 4.*

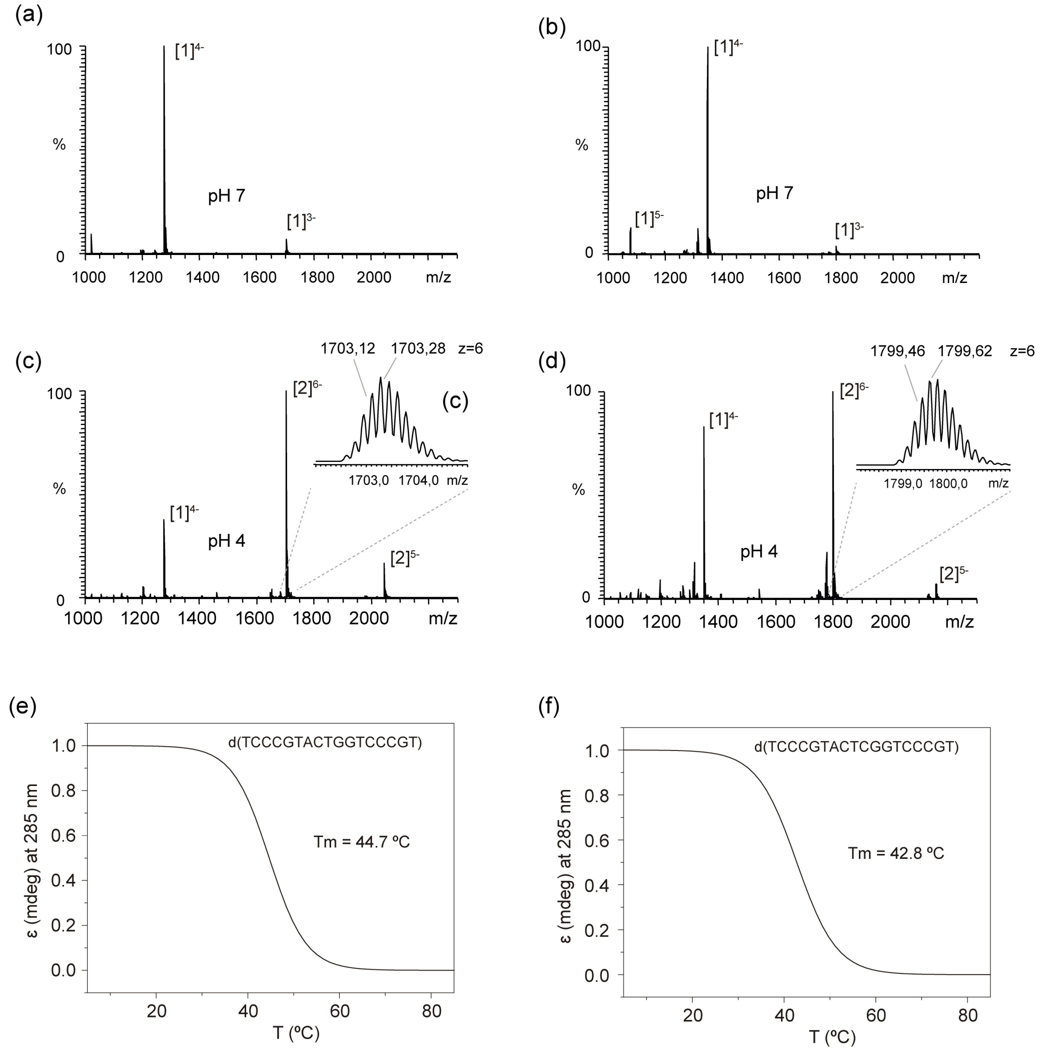

Figure S7. *Mass spectrometry data of undeca (left panels) and dodeca (right panels). Buffer conditions: 100 mM NH_4OAc, pH 7 (a, b); and 100 mM NH_4AcO, pH 4 (c, d). Zoom views of the dimer peaks showing the isotopic distribution are displayed in the insets of the panels e and f (separation between two consecutive ^{13}C isotopes in the isotopic distribution corresponds to m/z equals to 1/z, consequently in main isotopic distribution the z value is 6, and the mass is that of a dimer). Normalized CD melting curves of the undeca (e) and dodeca (f). Same conditions as Figure 3 c and d*.

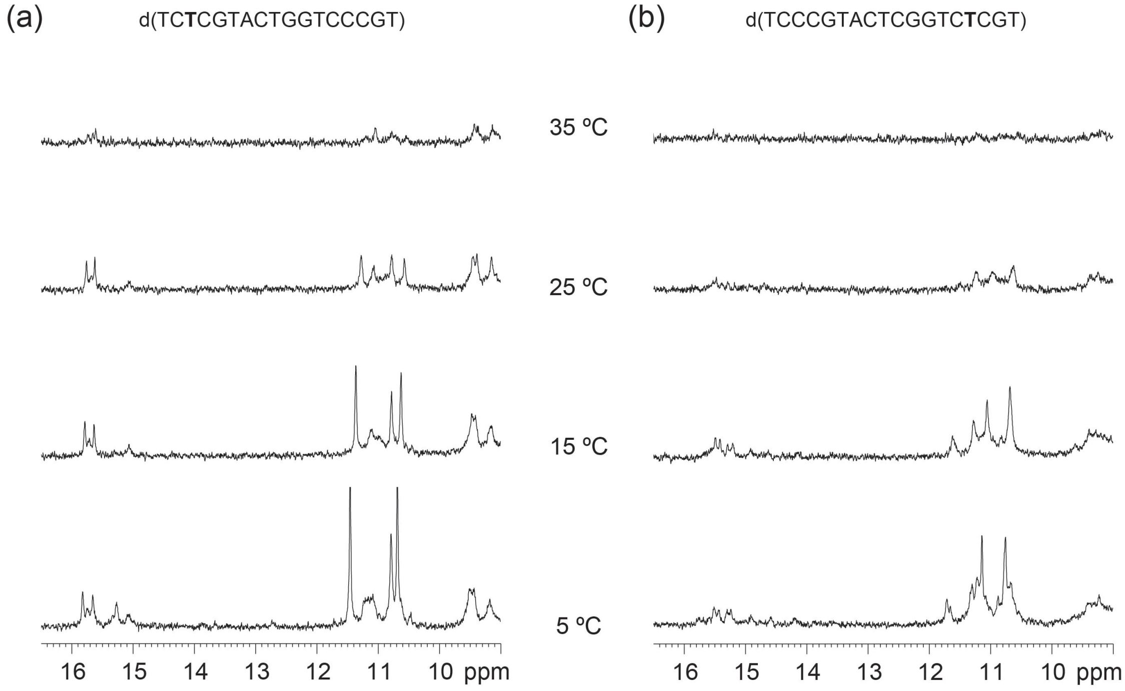

Figure S8. *Imino region of the NMR spectra of two control oligonucleotides, undecaT (a) and dodecaT (b). These oligonucleotides have the same sequence as undeca and dodeca but with a C→T mutation in the middle of the first and the second cytosine tracts, respectively (marked in bold). Spectra were recorded under the same buffer conditions as Supplementary Figure S6. The imino region of the spectra shows signals at 15-16 ppm that disappear at much lower temperature than the unmodified sequences (Supplementary Fig. S6). The destabilization effect of these single mutations is around 10ºC and 20ºC for the undeca and dodeca, respectively. This indicates that the C:C$^+$ base pairs are key elements for the stability of the structures formed.*

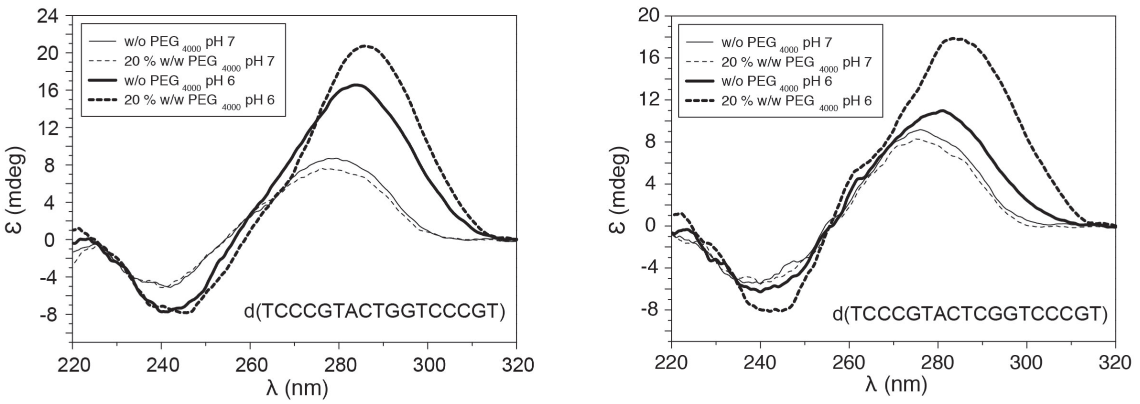

Figure S9. *CD spectra of the C-rich strands of undeca (left) and dodeca repeats (right) under molecular crowding conditions at different pH. Solid lines: CD spectra in the buffer 25 mM sodium phosphate, 100 mM NaCl; Dashed lines: CD spectra lines upon addition of 20%* PEG_{4000}*. Oligonucleotide concentration = 100 μM. Spectra were recorded at T= 5ºC.*

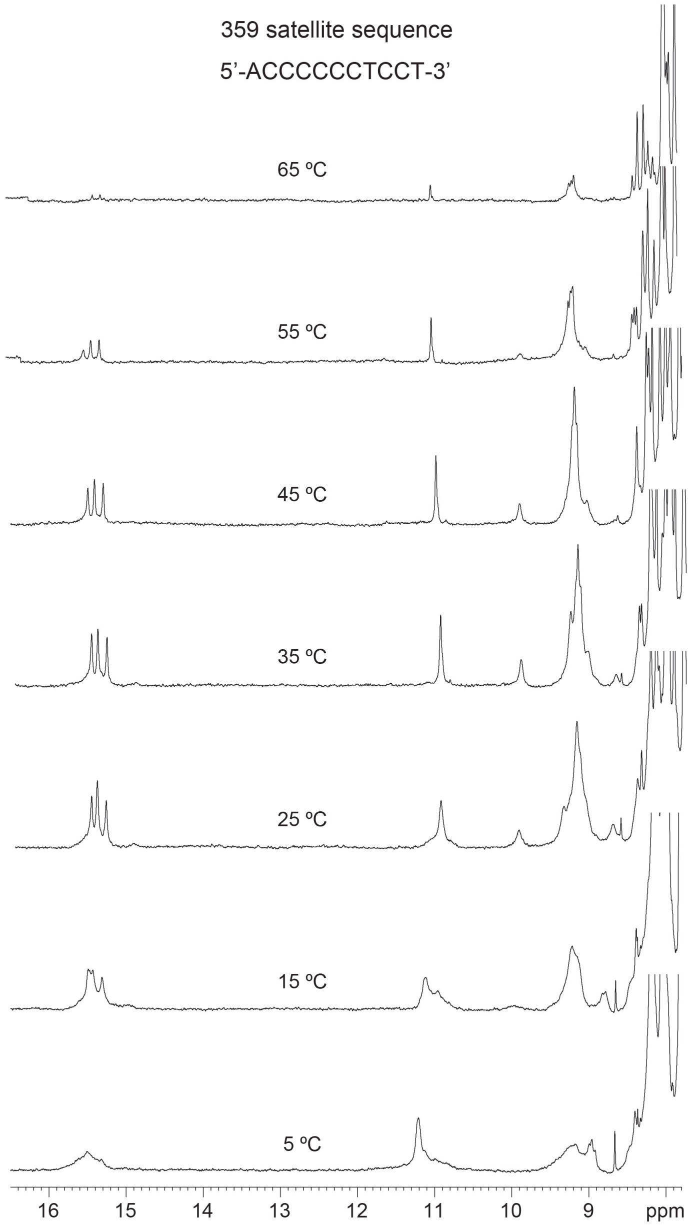

Figure S10. *Imino region of the NMR spectra (at different temperatures) of the C-rich region found at the 359 satellite DNA. The spectra show sharp imino signals around 15-16 ppm, which are characteristic features of i-motif formation. Buffer conditions: 25 mM NaPi, 100 mM NaCl pH=4.0. Oligo concentration: 0.6 mM*.

References

1 Crooks, G. E., Hon, G., Chandonia, J. M. & Brenner, S. E. WebLogo: a sequence logo generator. *Genome Res.* **14**, 1188-1190, (2004).
